# Use of CMR-based wave intensity analysis to demonstrate abnormalities in the aorta, the ventricle and ventriculo-arterial coupling: Comparison between patients with complete transposition of the great arteries (TGA), following palliation with atrial switch and arterial switch operations, and normals

**DOI:** 10.1186/1532-429X-15-S1-P293

**Published:** 2013-01-30

**Authors:** Giovanni Biglino, Hopewell Ntsinjana, Carla Plymen, Alessandro Giardini, Graham Derrick, Silvia Schievano, Andrew M Taylor

**Affiliations:** 1 Centre for Cardiovascular Imaging, Institute of Cardiovascular Science, University College London, London, UK; 2 Cardiorespiratory Unit, Great Ormond Street Hospital for Children, NHS Trust, London, UK

## Background

Long-term survivors of transposition of the great arteries (TGA) palliated with atrial switch (Senning or Mustard) operation or repaired with the arterial switch operation (ASO) are prone to complications. Aortic media has been shown to be structurally abnormal, even with unrepaired lesion, suggesting a congenital structural deficit. Progressive aortic root dilatation and reduced ascending aorta distensibility are frequent [1], following both types of repair. In this light, we aim to non-invasively compare the two systemic ventricles assessing the subclinical hemodynamic burdens of atrial and arterial switch operations using CMR-derived wave intensity analysis.

## Methods

54 cases were analysed, 18 healthy controls, 18 atrial switches and 18 arterial switches (Table 1). The phase-contrast MR flow sequence at the level of the ascending aorta was used for calculating wave speed (*c*) and wave intensity, using an in-house written plug-in (OsiriX, Pixmeo, Geneva) [2]. Knowledge of *c* from simultaneous changes in area and velocity at one location yielded aortic distensibility (D = 1/ρ*c*^2^), where ρ = density of blood. Wave intensity is a hemodynamic index evaluating the working condition of the heart in relation to the arterial network. From the calculated wave intensity patterns, the peaks of the forward compression wave (FCW) in early systole and forward expansion wave (FEW) in late systole were measured, as indicators of ventricular contractility and diastolic time constant τ, respectively [3].

**Table 1:** Characteristics of the study population

Variable	Controls	Atrial switch	Arterial switch
Patients (n)	18	18	18
Male/Female	12/6	13/5	9/9
Age (years)	15 ± 2	30 ± 6 *	15 ± 3 ^§^
BSA (m^2^)	1.7 ± 0.2	1.9 ± 0.2 *	1.7 ± 0.4 ^§^
EF (%)	69 ± 5	56 ± 7 *	66 ± 5 ^§^
iEDV (mL/m^2^)	75 ± 13	87 ± 22 *	86 ± 14 *
Aortic area (cm^2^)	3.6 ± 1.1	5.9 ± 1.5 *	7.3 ± 2.3 *^,§^

BSA = body surface area; EF = ejection fraction; iEDV = indexed end-diastolic volume. * indicates p<0.05 with respect to the control cohort; ^§^ indicates p<0.05 with respect to the atrial switch cohort

## Results

**Figure 1:**
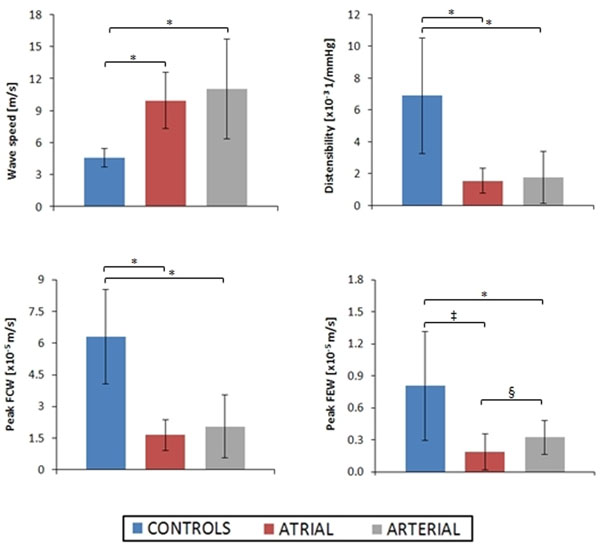
Summary of results indicating differences in wave speed, distensibility, peak forward compression wave (FCW) and forward expansion wave (FEW) between the groups. * indicates p<0.001, ‡ indicates p<0.005 and § indicates p<0.05.

Controls had higher ejection fraction and lower indexed end diastolic volume than both atrial and arterial switches. CMR-derived dimensions indicated that controls have a smaller aortic cross-sectional area than both atrial and arterial switches, with the latter larger than atrial switches. Our analysis indicated significant differences in *c*, and hence in D, between controls and TGA subjects. Also, the FCW peak was lower for both switches compared to controls. On the other hand, the FEW was lower in atrial switches than in controls, but interestingly in arterial switches it was higher than atrial switches. These results are summarised in Figure 1.

## Conclusions

CMR-derived wave intensity analysis highlighted differences in distensibility and ventriculo-arterial coupling between controls and TGA patients. Our method suggests the presence of an intra-operational difference between switch type in terms of diastolic behaviour (reduced FEW).

## Funding

National Institute of Health Research UK (NIHR); Fondation Leducq; Commonwealth Scholarships; Royal Academy of Engineering and EPSRC.
